# Untargeted Phytochemical Profiling, Antioxidant, and Antimicrobial Activities of a Tunisian *Capsicum annuum* Cultivar

**DOI:** 10.3390/molecules28176346

**Published:** 2023-08-30

**Authors:** Yossri Grojja, Hafedh Hajlaoui, Simon Vlad Luca, Jouda Abidi, Krystyna Skalicka-Woźniak, Sami Zouari, Mohamed Bouaziz

**Affiliations:** 1 Laboratory of Electrochemistry and Environment, National School of Engineers of Sfax, University of Sfax-Tunisia, B.P “1173”, Sfax 3038, Tunisia; grojja3@gmail.com (Y.G.); joudaabidi55@gmail.com (J.A.); 2 Faculty of Sciences and Technology of SidiBouzid, University of Kairouan, Campus University Agricultural City, Sidi Bouzid 9100, Tunisia; bio.hafedh@gmail.com; 3 Laboratory of Plant-Soil-Environment Interactions, LR21ES01, Faculty of Sciences of Tunis, University of Tunis EL Manar, Tunis 2092, Tunisia; 4 Biothermodynamics, TUM School of Life Sciences, Technical University of Munich, 85354 Freising, Germany; vlad.luca@tum.de; 5 Department of Pharmacognosy and Phytotherapy, Grigore T. Popa University of Medicine and Pharmacy, 700115 Iasi, Romania; 6 Department of Chemistry of Natural Products, Medical University of Lublin, 20-093 Lublin, Poland; 7 Laboratory of Medicinal and Environmental Chemistry, High Institute of Biotechnology of Sfax, University of Sfax, Sfax 3038, Tunisia; sami.zouari@enis.tn; 8 Higher Institute of Biotechnology of Sfax, University of Sfax, B.P “1175”, Sfax 3038, Tunisia

**Keywords:** antimicrobial, antioxidant, *Capsicum annuum*, polyphenols and flavonoids content, RP-HPLC-DAD-QTOF-MS/MS

## Abstract

Peppers are among the spices possessing a wide plethora of biological properties due to their excellent supply of health-related metabolites. *Capsicum annuum* L. (Solanaceae) is cultivated throughout Tunisia, and there is a shortage of information on the identification of the secondary metabolites in the seeds of this species as well as on their biological activities. In the present work, we intended to undertake a chemical characterization of the bioactive compounds from the hydro-methanolic seed extract of *C. annuum* as well as an evaluation of its broad spectrum of antimicrobial and antioxidant activities. The chemical profile was evaluated by RP-HPLC-DAD-QTOF-MS/MS, whereas the total phenol and flavonoid content, antioxidant, and antimicrobial activities were determined in in vitro assays. In this work, 45 compounds belonging to various phytochemical classes, such as organic acids (2), phenolic compounds (4 phenolic acids and 5 flavonoids), capsaicinoids (3), capsianosides (5), fatty acids (13), amino acids (1), sphingolipids (10), and steroids (2) were identified in the hydro-methanolic seed extract of *C. annuum*. The phenolic and flavonoid content (193.7 mg GAE/g DW and 25.1 mg QE/g DW, respectively) of the *C. annuum* extract correlated with the high antiradical activity (IC_50_ = 45.0 µg/mL), reducing power (EC_50_ = 61.3 µg/mL) and chelating power (IC_50_ = 79.0 µg/mL) activities. The hydro-methanolic seed extract showed an important antimicrobial activity against seven bacterial and four fungal strains. In fact, the inhibition zones (IZs) for bacteria ranged from 9.00 ± 1.00 mm to 12.00 ± 0.00 mm; for fungi, the IZs ranged from 12.66 ± 0.57 mm to 13.66 ± 0.57 mm. The minimal inhibition concentration and minimal bactericidal concentration values showed that the extract was more effective against fungi than bacteria.

## 1. Introduction

The genus *Capsicum* belongs to the Solanaceae family and consists of about 27 species [1]. It was considered a typical American genus, and several species of *Capsicum* were cultivated for thousands of years by pre-Columbian civilizations [2]. *Capsicum* species are popular vegetables and spices grown worldwide, especially in tropical and subtropical countries [3]. Based on the taxonomic identification of these plant species (peppers), four varieties are recognized, namely, *Capsicum annuum* var. abbreviatum, *Capsicum annuum* var. *acuminatum*, *Capsicum annuum* var. *grossum*, and *Capsicum frutescens* var. *baccatum* [4].

In Tunisia, pepper cultivars are mainly represented by *C. annuum* species, with a number of chili pepper landraces [5] cultivated throughout the country, in addition to a few *C. frutescens* cultivars [6,7]. Interest in peppers has increased not only because of their high nutritional value as a vegetable, food ingredient, and coloring agent in the food industry [8,9,10] but also for their cosmetic and medical uses [11,12]. Indeed, pepper is recognized as an excellent source of health-related metabolites, such as ascorbic acid (vitamin C), carotenoids (provitamin A), tocopherols (vitamin E), polyphenols, and capsaicinoids [13]. Polyphenols, including flavonoids, are secondary metabolites that are abundant in plants. It is known that these components play a protective role against pathogens and UVB light in the interaction between plants and the environment [14,15]. Therefore, polyphenolic compounds have attracted a great deal of attention, since they act as antioxidants and protect the human body from oxidative stress, which is the main cause of different degenerative processes. Thus, consuming fruits and vegetables is reversely correlated with the development of chronic diseases [16]. Due to their health benefits, polyphenols have gained a great deal of attention, especially in vegetables such as peppers that are consumed in large amounts worldwide. Thus, peppers are among the vegetables that provide a rich source of various bioactive compounds with potential biological properties. For example, the appreciable amount of phenols and flavonoids in the ethanolic extracts of *C. annuum* contributed to their antiradical activities [17]. In another study, methanolic extracts from *C. annuum* were reported to inhibit 4-hydroxy-2-nonenal- and H_2_O_2_-induced DNA damage [18].

On the other hand, only a few studies have shown that *C. annuum* has antimicrobial activities against different microorganisms [19,20,21]. Koffi-Nevry et al. [19] demonstrated that *C. annuum* exhibited inhibitory activity against *Escherichia coli*, *Vibrio cholerae*, *Staphylococcus aureus*, and *Pseudomonas aeruginosa*. Moreover, Sree Sandhya and Vijayakumar [20] reported that an ethanolic extract of *C. annuum* var. *glabriusculum* inhibited two microbial strains (*Staphylococcus aureus* and *Streptococcus mutans*). More recently, aqueous, ethanolic, and ethyl acetate extracts of *C. annuum* were tested against several pathogenic fungal strains (*Alternria* sp., *Penicillium* sp., *Fusarium* sp., *Aspergillus flavus*, and *Aspergillus niger*). It was demonstrated that these extracts inhibited all the strains used except for *Aspergillus niger* [21].

Furthermore, there is a shortage of information on the identification of secondary metabolites in the seeds of the Tunisian *C. annuum* cultivar as well as on their antioxidant activities. To the best of our knowledge, only one study was carried out by LC-MS/MS on the identification of phenolic compounds in the ethanolic seed extract of three types of Australian-grown bell peppers (green, red, and yellow) and the estimation of their antioxidant potential [22]. As a valorization of the by-products of *C. annuum* (seeds) discarded by a Tunisian harissa factory located in the northeast of Tunisia (Cap Bon), this study was geared towardsa chemical characterization of the bioactive compounds from a hydro-methanolic seed extract of *C. annuum* as well as an evaluation of its broad spectrum of antioxidant and antimicrobial activities.

## 2. Results and Discussion

### 2.1. Metabolite Characterization of C. annuum Seed Extract by RP-HPLC-DAD-QTOF-MS/MS

**Figure 1:**
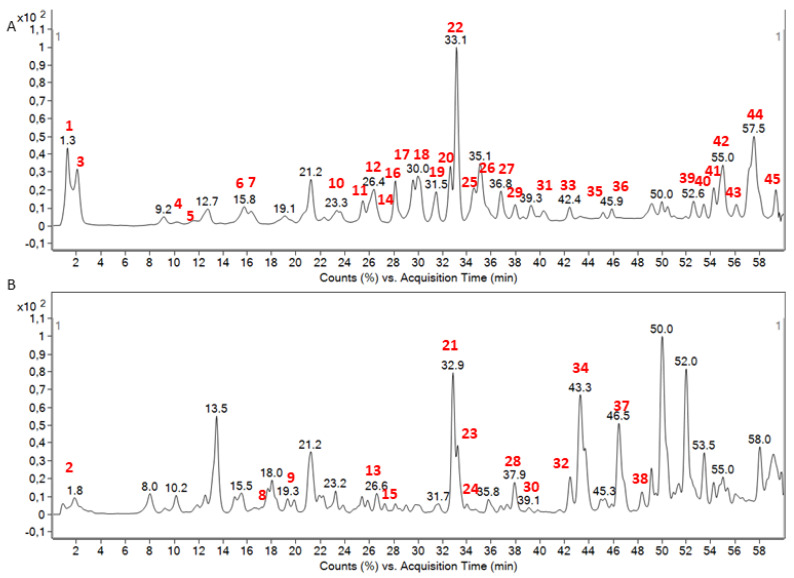
Base peak chromatogram in the negative ionization mode (**A**); and positive ionization mode (**B**) of the RP-HPLC-DAD-QTOF-MS/MS analysis of the Tunisian *C. annuum* hydro-methanolic seed extract; with the red color as the peak number, according to Table 1.

In the present work, the metabolite profiling of the hydro-methanolic extract of *C. annuum* seeds was established using RP-HPLC-DAD-QTOF-MS/MS. Figure 1A,B presents the base peak chromatogram (BPC) profiles of the analyzed extract in both negative (A) and positive (B) ionization modes. The peak numbers are given following the elution order (retention time, T_R_), as listed in Table 1. Table 1 also summarizes the experimental *m*/*z* of the precursor ion, molecular formula, and main MS/MS fragments in the negative or positive ionization modes. The identified compounds were classified into various groups, as follows: organic acids (2), phenolic compounds (4 phenolic acids and 5 flavonoids), capsaicinoids (3), capsianosides (5), fatty acids (13), amino acids (1), sphingolipids (10), and steroids (2). Using the abovementioned method, it was possible to identify 45 compounds belonging to various phytochemical classes in the *C. annuum* hydro-methanolic seed extract.

#### 2.1.1. Organic Acids

In the polar region of the BPC (Figure 1A), two organic acids with low molecular masses were identified in the hydro-methanolic extract of *C. annuum* seeds (negative ionization mode). Galactonic/gluconic acid (peak **1**, C_6_H_12_O_7_) was detected as the precursor ion [M−H]^−^ at *m*/*z* 195.0563. Its MS/MS spectra showed fragment ions at *m*/*z* 177.0444 [M–H–H_2_O]^−^, 159.0358 [M–H–2H_2_O]^−^, 129.0233 [M–H–2H_2_O–CH_2_O]^−^. In addition, RP-HPLC-DAD-QTOF-MS was helpful in the identification of citric acid (peak **3**) with the precursor ion [M−H]^−^ at *m*/*z* 191.0241 and the molecular formula C_6_H_8_O_7_. Moreover, the structure of this organic acid was confirmed by the presence of the diagnostic fragment ion at *m*/*z* 111.0409, corresponding to [M–H–CO_2_–2H_2_O]^−^ in its MS/MS spectra. Citric acid was previously described in the literature; its fragmentation patterns were in accordance with previous studies [23,24,25]. In fact, citric acid was identified in many Balkan pepper accessions [25] as well as in a population of wild Piquin Chili (C. *annuum* var. *glabriusculum*) [26].

#### 2.1.2. Phenolic Compounds

**Figure 2:**
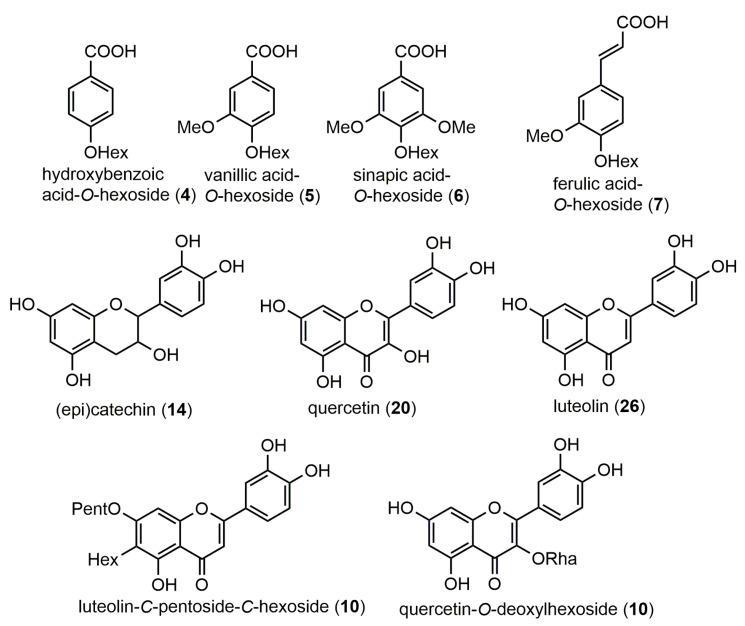
Potential structures of phenolic compounds identified in the Tunisian *C. annuum* hydro-methanolic seed extract.

Two sub-classes of phenolic compounds were characterized in the negative ionization mode, namely, phenolic acids and flavonoids (Figure 2). In the case of phenolic acids, four compounds were identified in the BPC (retention time range 10.2–16.5 min). Peak **4**, with the pseudo-molecular ion [M−H]^−^ at *m*/*z* 299.0845 (C_13_H_16_O_8_), was assigned to hydroxybenzoic acid-*O*-hexoside. It is worth mentioning that this compound was previously reported in peppers such as *C. chinense* [27] and *C. annuum* [28]. A derivative of this compound, namely, vanillic acid-*O*-hexoside (peak **5**), was shown to possess the precursor ion at *m*/*z* 329.0943 (C_13_H_16_O_8_), indicating the presence of an additional methoxy group. Furthermore, two hydroxycinnamic acid derivatives were assigned, sinapic acid-*O*-glucoside (peak **6**, [M−H]^−^ at *m*/*z* 385.1938) and ferulic acid-*O*-hexoside (peak **7**, [M−H]^−^ at *m*/*z* 355.1106). The MS/MS spectra of the latter compound showed two abundant fragment ions at *m*/*z* 193.0542 [M–H–hexosyl]^−^ and 175.0440 [M–H–hexosyl–H_2_O]^−^; these fragments are in agreement with those reported by Leng et al. [22]. Both compounds were previously detected in *Capsicum* peppers [27,29].

The second class of phenolic compounds detected in the present study was flavonoids (Table 1). They are among the most important phenolic compounds distributed mainly in the *Capsicum* genus, such as *C. annuum* var. *glabriusculum* [26] and *C. chinense* [27]. Five compounds belonging to various flavonoid classes (flavones, flavanols, and flavonols) were identified in the seeds of *C. annuum*. In fact, two luteolin derivatives were detected, namely luteolin-*O*-pentoside-*C*-hexoside (peak **10**, [M−H]^−^ at *m*/*z* 579.1437) and its free aglycon luteolin (peak **26**, [M−H]^−^ at *m*/*z* 285.0407). The glycosylated derivative showed, in its MS/MS spectrum, the loss of the pentosyl group (−132 Da), indicating the *O*-glycosylation with pentose. However, the subsequent fragment ion at *m*/*z* 327.0554 was obtained from the previous ion by the diagnostic removal of a group with 120 Da, indicative of a *C*-glycosylation with a hexose unit. Luteolin and its derivative were previously reported in *Capsicum* peppers by several authors [27,29,30]. Three other flavonoid peaks were detected in the hydro-methanolic extract of *C. annuum* seeds. (Epi)catechin (peak **14**), with the precursor ion [M−H]^−^ at *m*/*z* 289.1038 (C_15_H_14_O_6_), showed diagnostic fragments at *m*/*z* 245.0865 [M–H–CO_2_]^−^ and 205.0511 [M–H–C_4_H_4_O_2_]^−^, which were consistent with previous studies [22,31,32]. This compound was previously found in the ethanolic seed extracts of three types of Australian-grown bell peppers [27]. Quercetin-*O*-deoxyhexoside (peak **11**) showed a precursor ion [M−H]^−^ at *m*/*z* 447.1011 (C_21_H_20_O_11_), which was fragmented to *m*/*z* 271.0443, 255.0404, and 179.0086, as mentioned by Jeong et al. [33]. Quercetin (peak **20**) exhibited a precursor ion [M−H]^−^ at *m*/*z* 301.0383 (C_15_H_10_O_7_) which generated the specific fragments at *m*/*z* 273.0376 and 178.9983, as cited by Santos et al. [30] and Schelz et al. [34]. Previously, quercetin-3-O-rhamnoside was found in *C. annuum* fruits [27], whereas quercetin was reported in the ethanolic extract of *C. chinense* ripe fruits [30], as well as in the methanolic extract of the unripe fruit of *C. annuum* [33].

#### 2.1.3. Capsaicinoids

**Figure 3:**
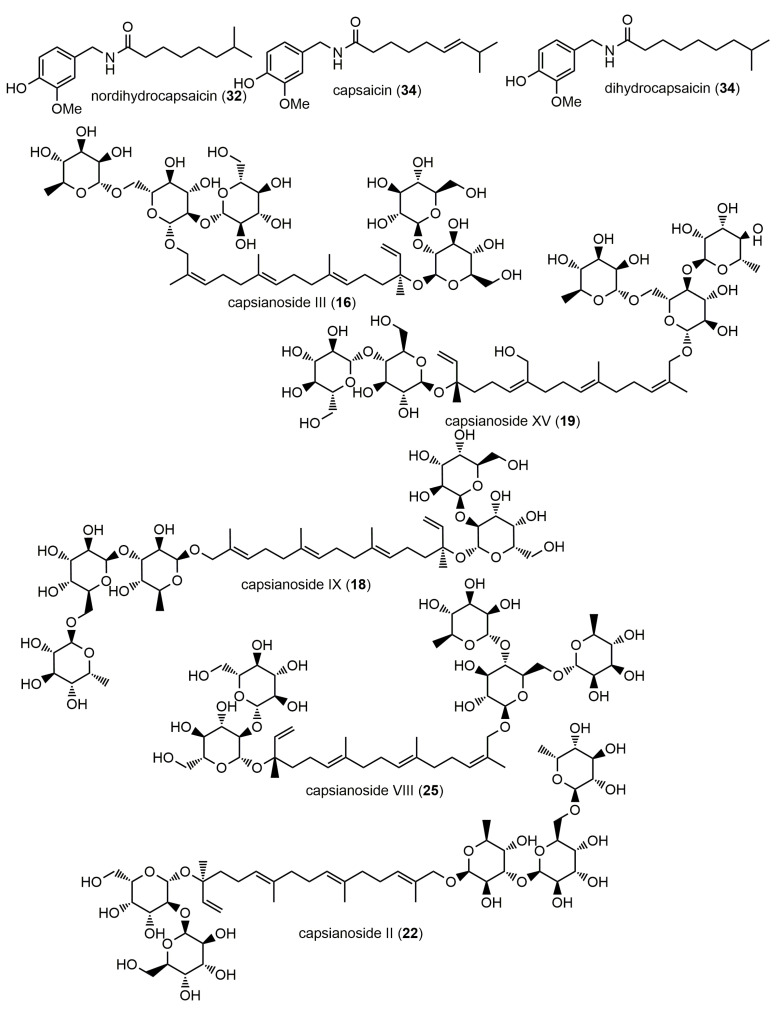
Potential structures of capsaicinoids and capsianosides identified in the Tunisian *C. annuum* hydro-methanolic seed extract.

All capsaicinoids (amides) were characterized in the positive ionization mode, which showed the protonated molecule ions [M+H]^+^ of norhydrocapsaicin (peak **32**, *m*/*z* at 294.2072), capsaicin (peak **34**, *m*/*z* at 306.2069), and dihydrocapsaicin (peak **37**, *m*/*z* at 308.2229), as shown in Table 1. Capsaicin and dihydrocapsaicin, which differ only by a double bond on their lateral carbonic chain (Figure 3), showed identical fragments at *m*/*z* 137.0579. However, capsaicin and dihydrocapsaicin showed a characteristic fragment at *m*/*z* 182.1524 and 184.1668, respectively. The importance of these compounds is related to several factors. On the one hand, they are considered to be the main active ingredients in *C. annuum* seeds [28]; hence, they are valuable pharmaceutical ingredients. On the other hand, they are responsible for the hot taste of peppers [29,30].

#### 2.1.4. Capsianosides

Capsianosides are a large group of diterpenic glycosides characteristic of *Capsicum* peppers [35]. In the current study, five capsianoside derivatives (Figure 3) were putatively labeled by RP-HPLC-DAD-QTOF-MS/MS (negative ionization mode) in the hydro-methanolic extract of *C. annuum* seeds (Table 1), namely, capsianoside III (peak **16**, C_50_H_84_O_26_), capsianoside IX (peak **18**, C_44_H_74_O_21_), capsianoside XV (peak **19**, C_50_H_84_O_26_), capsianoside II (peak **22**, C_50_H_84_O_25_), andcapsianoside VIII (peak **25**, C_50_H_84_O_25_). In their MS/MS spectra, the removal of the corresponding sugar units was diagnostically observed. For instance, in the case of capsianosides III and XV with [M−H]^−^ at *m*/*z* 1099.5317 and 1099.5379, respectively, the following fragment ions were noticed at *m*/*z*: 937.4759 [M–hexosyl–H]^−^, 775.4192 [M–2hexosyl–H]^−^, 629.3666 [M–2hexosyl–deoxyhexosyl–H]^−^, and 467.2929 [M–3hexosyl–deoxyhexosyl–H]^−^.

#### 2.1.5. Fatty Acids

Thirteen oxygenated fatty acids were detected in the negative ionization mode in the non-polar region of the chromatogram (retention times from 34.5 to 59.3 min). Practically, all compounds were derived from octadecanoic acid (C18), with the differences residing in the number of oxygenated functions and double bonds; their *m*/*z* values ([M−H]^−^) ranged from 293.2203 and 331.2124. Thus, acids with one double bond (e.g., trihydroxyoctadecenoic acid, dihydroxyoctadecenoic acid), two double bonds (e.g., hydroperoxyoctadecadienoic acid, hydroxyoctadecadienoic acid) and three double bonds (e.g., hydroxyoctadecatrienoic acid) were putatively identified in the *C*. *annuum* seed extract (Table 1). Fatty acids reported in our study were also previously documented in *C. chinense* extracts [36].

#### 2.1.6. Amino Acids and Amino Alcohols (Sphingolipids)

One amino acid and 10 amino alcohols (sphingolipids) were tentatively assigned by RP-HPLC-DAD-QTOF-MS/MS (positive ionization mode) in the hydro-methanolic extract of *C. annuum* seeds (Table 1). The precursor ion [M+H]^+^ at *m*/*z* 294.1539 (C_12_H_23_NO_7_) and MS/MS fragment ions at *m*/*z* 258.1321, 230.1388, 144.0990, and 114.0984 were in agreement with the spectrometric data reported by Menezes et al. [36] for *N*-fructosyl(iso)leucine (peak **2**).

**Figure 4:**
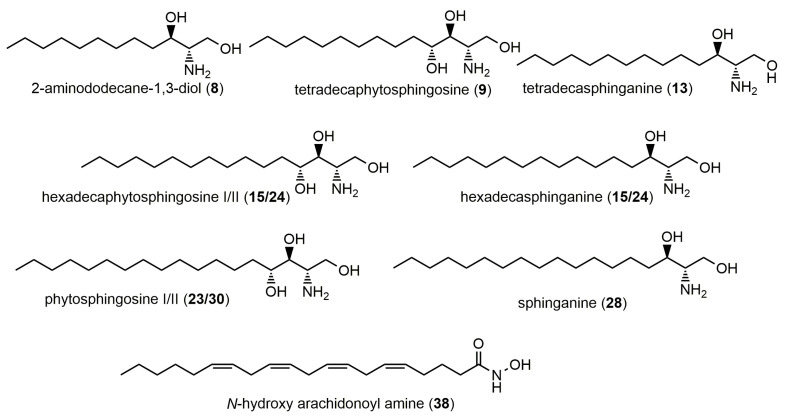
Potential structures of sphingolipids identified in the Tunisian *C. annuum* hydro-methanolic seed extract.

Sphingolipids are structurally derived from fatty alcohols with one vicinal amino group and several additional hydroxyl groups (Figure 4). In the *C. annuum* extract, compounds with C12 (2-aminododecane-1,3-diol), C14 (tetradecaphytosphingosine, tetradecasphinganine), C16 (hexadecaphytosphingosine, hexadecasphinganine), C18 (phytosphingosine, sphinganine), and C20 (*N*-hydroxy arachidonoyl amine) atoms were tentatively identified. Since each derivative differed from the previous one by two carbon atoms and/or an extra hydroxyl group, these structural differences were easily noticeable in the MS spectra, which allowed for the annotation of the molecular formulas. The MS/MS fragmentation patterns of these amino alcohols generally show the characteristic loss of a hydroxyl group [M–H–H_2_O]^+^. Similar sphingolipids, such as sphinganine 1-phosphate, phytosphingosine, sphinganine 1-phosphate, sphinganine, 2-aminoicosane-1,3-diol, hexadecasphinganine, and soyacerebroside, were previously reported in *C. annuum* by Guevara et al. [37] and Cervantes-Hernandez et al. [38].

#### 2.1.7. Steroids

Lastly, two steroidal glycosidic saponins were putatively annotated in the analyzed extract. Capsicoside A (peak **12**, [M−H]^−^ at *m*/*z* 1421.6593) showed in its MS/MS spectra the successive loss of the sugar units bound to its aglycon, as follows: *m*/*z* 1259.5962 [M–hexosyl–H]^−^, 1097.4411 [M–2hexosyl–H]^−^, 935.4233 [M–3hexosyl–H]^−^, 773.4179 [M–4hexosyl-H]^−^. Similarly, protodegalactotigonin (peak **17**, [M−H]^−^ at *m*/*z* 1213.6011) presented an MS/MS fragment ion at *m*/*z* 1081.5401, 919.5061, and 757.4474, derived by the neutral successive cleavage of one pentose and two hexose units. These two steroidal glycosides were previously reported in *C. annuum* by Yahara et al. [39].

### 2.2. Total Phenolic Content, Flavonoid Content, and Antioxidant Activities of C. annuum Seed Extract

Table 2 presents the results for the polyphenol and flavonoid content of the *C. annuum* hydro-methanolic extract, as well as its antioxidant activities. In our study, we used three complementary methods for antioxidant activity determination, such as DPPH, reducing power, and chelating power assay [40]. As compared to BHT (11.5 µg/mL), vitamin C (37.0 µg/mL), and EDTA (32.5 µg/mL), the moderate polyphenols and flavonoids content (193.7 mg GAE/g DW and 25.1 mg QE/g DW, respectively) of the *C. annuum* hydro-methanolic extract necessarily show relatively high antiradical activity (IC_50_ = 45.0 µg/mL), reducing power (EC_50_ = 61.3 µg/mL) and chelating power (IC_50_ = 79.0 µg/mL) activities. Compared to other varieties of *C. annuum* species [4], the hydro-methanolic seed extract of *C.annuum* is relatively low in its content of polyphenols and flavonoids. In fact, the polyphenols content of the ethanolic extract of mature fruits of *C. annuum* var. *abbreviatum*, *C. annuum* var. *acuminatum*, *C. annuum* var. *grossum* ranged from 200.70 to 272.74 mg GAE/g DW, whereas the flavonoid content of the same extracts varied between 1223.71 and 1630.53 mg QE/g DW [4]. Moreover, the DPPH radical scavenging activities of this extract changed in the order: *C. annuum* var. *abbreviatum* > *C. annuum* var. *acuminatum* > *C. annuum* var. *grossum* [4].

### 2.3. Antimicrobial Activity of C. annuum Extract

The data presented in Table 3 demonstrate the inhibitory diameter zones (IZs) for both bacterial and fungal strains. Specifically, for the hydro-methanolic seed extract, the IZs range from 9.00 ± 1.00 mm to 12.00 ± 0.00 mm for bacterial strains and from 12.66 ± 0.57 mm to 13.66 ± 0.57 mm for fungal strains. On the other hand, for the reference antibiotic gentamycin, the IZs span from 21.33 ± 0.58 mm to 27.67 ± 1.53 mm for bacterial strains, and for amphotericin B, the range is 16.00 ± 0.00 mm to 18.00 ± 0.00 mm for fungal strains.

Upon conducting the statistical analysis, it was observed that the hydro-methanolic seed extract of *C. annuum* exhibited lower efficacy than the reference antibiotics. This suggests that the extract may not be as potent in inhibiting the growth of bacterial and fungal strains. Furthermore, the statistical analysis (*p* < 0.05) of the inhibition diameters indicates significant differences in the resistance of the strains against the hydro-methanolic seed extract. Interestingly, the Gram-negative bacteria displayed the highest sensitivity to the extract, as evidenced by the larger inhibitory zone observed for this group. Overall, these findings suggest that the hydro-methanolic seed extract of *C. annuum* may have limited antimicrobial effectiveness, particularly against Gram-negative bacteria. Further investigations and studies may be warranted to better understand the potential applications and limitations of this extract as an antimicrobial agent.

The quantitative method (Table 3) reveals the MIC and MBC values for the hydro-methanolic seed extract. The MIC values range from 0.15 mg/mL (for *E. faecalis* and *M. luteus*) to 1.875 mg/mL (for *S. epidermidis*, *B. cereus*, *L. monocytogenes*, and *S. typhimurium*), while the MBC values vary from 1.875 mg/mL (for *M. luteus*) to 7.50 mg/mL (for *E. coli*). In contrast, for the fungal strains, the MIC and MFC values were comparatively lower. This outcome indicates a higher level of sensitivity to the *C. annuum* hydro-methanolic seed extract among the tested bacterial strains. However, when comparing these values, it becomes apparent that the antibiotics or antifungal agents seem to exhibit higher activity than the hydro-methanolic seed extract.

The MBC/MIC and MFC/MIC ratios were utilized to gain insights into the antimicrobial effect of the hydro-methanolic seed extract. Based on the classification provided by Schaechter et al. [41] and Soro et al. [42], a ratio greater than four indicates a bacteriostatic or fungistatic effect, whereas a ratio equal to or lower than four suggests a bactericidal or fungicidal effect. Upon examining the ratios presented in Table 3, it becomes evident that the hydro-methanolic seed extract displayed bactericidal and fungicidal properties against all the tested strains. This finding indicates that the extract is effective in not only inhibiting the growth (bacteriostatic/fungistatic) but also in killing the bacteria and fungi (bactericidal/fungicidal) at or below the concentrations tested.

The observed antimicrobial activity of the hydro-methanolic seed extract can be attributed to its chemical composition, particularly its high content of phenolic compounds (193.7 ± 3.1 mg GAE/g). The RP-HPLC-DAD-QTOF-MS/MS analysis of the Tunisian *C. annuum* hydro-methanolic seed extract revealed the presence of several compounds known for their biological activity, which likely contribute to the extract’s antimicrobial properties. Some of these bioactive compounds include citric acid, hydroxybenzoic acid-*O*-hexoside, ferulic acid-*O*-hexoside, quercetin-*O*-deoxyhexoside, (epi)catechin, quercetin, luteolin, and capsaicin. Each of these compounds was previously studied for its potential antimicrobial effects, and their presence in the extract may synergistically enhance its overall antimicrobial activity against the tested bacterial and fungal strains. Citric acid is known for its acidic and chelating properties, which can inhibit bacterial growth [19,43,44,45]. Hydroxybenzoic acid-*O*-hexoside and ferulic acid-*O*-hexoside are phenolic compounds with reported antimicrobial and antioxidant properties [46,47]. Quercetin-*O*-deoxyhexoside, (epi)catechin, quercetin, and luteolin are flavonoids known for their antibacterial and antifungal activities. Capsaicin, a compound found in chili peppers, has also demonstrated antimicrobial properties against various pathogens [48].

The combined presence of these bioactive compounds in the hydro-methanolic seed extract likely contributes to its broad-spectrum antimicrobial efficacy against both bacterial and fungal strains. It is important to note that the synergistic interactions among these compounds and their individual concentrations can significantly influence the overall antimicrobial effectiveness of the extract.

## 3. Materials and Methods

### 3.1. Plant Material and Extraction Procedure

Mature fruits of *Capsicum annuum* were harvested from a farmer’s field in the CHAFFAR region (MAHRES, Sfax, Tunisia) at the end of September 2020. The fruits were air-dried at room temperature (25 °C) for 15 days. Then, the corresponding seeds were extracted from dried fruit and crushed into fine powder. Regarding the extraction procedure, 10 g of powder seeds were put in an amber glass bottle and homogenized in 100 mL of a mixture of methanol/water 80:20 (*v*/*v*) using an ultrasonic bath for 30 min at a power of 2500 W. After filtration with Whatman filter paper No. 42 (125 mm), the extract was evaporated with a rotary evaporator under vacuum at 40 °C. Finally, the dry extract was obtained with a yield of 7.6% and was kept at −20 °C until future analysis.

### 3.2. RP-HPLC–DAD-QTOF-MS/MS Analysis

The *Capsicum annuum* seed extract was analyzed by RP-HPLC-DAD-QTOF-MS/MS using the procedures described by Ammar et al. [49]. The chromatographic analytical procedures were performed on an Agilent 1200 Series (Agilent Technologies, Palo Alto, CA, USA) LC system coupled to a DAD and an Agilent 6540 Ultra-High-Definition (UHD) Accurate-Mass QTOF with a Jet Stream dual ESI interface. The instrument was equipped with a vacuum degasser, a binary pump, an autosampler with a thermostat, and a column compartment. The separations were carried out on a Phenomenex Gemini RP-18 (100 mm × 2 mm; i.d. 3 μm; Phenomenex, Torrance, CA, USA) column maintained at 20 °C. The mobile phase consisted of 0.1% formic acid in water (solvent A) and 0.1% formic acid in acetonitrile (solvent B). The elution gradient was used as follows: 0 min, 1% B; 13 min, 25% B; 20 min, 25% B; 25 min, 40% B; 30 min, 60% B; 35 min, 100% B; 40 min, 100% B; post-time 12 min. The flow rate was 0.20 mL/min. The ultraviolet (UV) spectra were recorded from 190 to 600 nm. The sample was diluted with a methanol/water mix of 80:20 (*v*/*v*) prior to the injection of 10 μL sample volume. The MS analyses were carried out using the following operating conditions: drying nitrogen temperature at 350 °C with a flow of 12 L/min; nebulizer pressure 40 psi; sheath gas temperature 400 °C with a flow of 12 L/min; capillary voltage, skimmer, and radiofrequency voltages of 4000, 645 and 750 V, respectively. The spectra were acquired in negative and positive ionization modes over a mass-to-charge (*m*/*z*) range of 100 to 1000. The reference mass correction of the sample was performed with a continuous infusion of Agilent API TOF reference mixture (61969–85001). The data analysis was carried out with Mass Hunter Qualitative Analysis B.06.00 (Agilent Technologies) software that enabled the generation of the molecular formula with a mass accuracy limit of 5 ppm and an MS ≥ 80 (related to the contribution to mass accuracy, isotope abundance, and isotope). For the retrieval of the chemical structure information, some databases were consulted as follows: PubChem (http://pubchem.ncbi.nlm.nih.gov), ChemSpider (http://www.chemspider.com), SciFinderScholar (https://scifinder.cas.org), Reaxys (http://www.reaxys.com), Phenol-Explorer (www.phenol-explorer.eu) and KNApSAcK Core System (http://kanaya.naist.jp/knapsackjsp/top.html) (accessed on 5 August 2023).

### 3.3. Total Polyphenol Compounds Analysis

Total polyphenol compounds were determined colorimetrically with Folin–Ciocalteu’s reagent according to the method of Gargouri et al. [50], with some modifications. Briefly, 50 μL of the suitable sample dilution was added to 250 μL of the Folin–Ciocalteu reagent. The mixture was shaken before adding 500 μL of Na_2_CO_3_ (20%) solution, adjusting with distilled water to a final volume of 5 mL, and mixed thoroughly. After incubation of the mixture for 30 min at 25 °C in darkness, the absorbance versus a prepared blank was read at 727 nm. A standard curve of gallic acid was used. The total phenolic content of the extract was expressed as mg gallic acid equivalents per gram of dry weight (mg GAE/g DW) through a calibration curve with gallic acid. The calibration curve range was 0–160 μg/mL (R^2^ = 0.98). The sample was analyzed in three replicates.

### 3.4. Total Flavonoid Content Analysis

The total flavonoid content was determined with AlCl_3_ reagent, according to Mouhamadi et al. [51], with some modifications. An amount of 1 mL of the diluted sample (500 mg/L in methanol) was added to 4 mL of distilled water and 300 μL of the NaNO_2_ solution (50%) and mixed for 6 min before adding 300 μL of AlCl_3_ (10%). After 5 min, 2 mL of NaOH (1M) was added. The final volume was adjusted to 10 mL with distilled water and thoroughly mixed. The absorbance of the mixture was determined at 510 nm against the same mixture, without the sample, as a blank. The total flavonoid content was expressed as mg quercetin/g dry weight (mg QE/g DW) through a calibration curve of quercetin. The calibration curve range was 0–120 μg/mL (R^2^ = 0.99). The sample was analyzed in three replicates.

### 3.5. Antioxidant Activity

#### 3.5.1. DPPH Radical Scavenging Ability Assay

DPPH quenching ability of the hydro-methanolic extract was measured according to Bouaziz et al. [52]. Briefly, a volume of 500 μL of each sample at different concentrations (10 to 100 µg/mL) was added to 375 μL of 99% ethanol and 125 μL of DPPH solution (0.02% in ethanol) as the free radical source. The obtained mixtures were shaken and then incubated for 60 min in the dark at room temperature. The measurement of the scavenging capacity was carried out spectrophotometrically by controlling the decrease of absorbance at 517 nm. The DPPH, in its radical form, has an absorption band at 517 nm, which vanishes upon reduction by an antiradical compound. A low absorbance of the reaction mixture reveals high DPPH free radicalscavenging activity. BHT was used as a positive control, and the calculation of DPPH radicalscavenging activity was performed as follows:

$$\%\;\mathrm{scavenging}\;\mathrm{effect}=\frac{ADPPH-AE}{ADPPH}\;\times \;100$$

where AE denotes the absorbance of the solution when the sample extract is added at a specific level, and ADPPH is the absorbance of the DPPH solution.

The antiradical activity was expressed as IC_50_ (µg/mL), the extract dose required to cause a 50% inhibition.

#### 3.5.2. Reducing Power

The ability of the extract to reduce Fe^3+^ was assayed using the method described by Yildirim et al. [53]. Briefly, 1 mL of the hydro-methanolic extract at different concentrations was mixed with 2.5 mL of phosphate buffer (0.2M, pH 6.6) and 2.5 mL of 1% K_3_Fe(CN)_6_. The resulting mixture was incubated for 20 min at 50 °C. After the addition of 2.5 mL of 10% (*w*/*v*) trichloroacetic acid, the solution was manually shaken. Lastly, 2.5 mL of the supernatant solution was mixed with 2.5 mL of distilled water and 500 µL of 0.1% (*w*/*v*) ferric chloride. After 10 min, the absorbance was measured at 700 nm. The EC_50_ value (µg/mL) is the effective concentration at which absorbance was 0.5 for reducing power. BHT and Vitamin C were used as the positive control.

#### 3.5.3. Chelating Effect on Ferrous Ions

The iron chelating activity of the different samples was estimated according to the protocol described by Dhouibi et al. [54], with slight modifications. Indeed, 50 μL of 2mM FeCl_2_, 4H_2_O was added to 100 μL of the extract at different concentrations (10 to 100 µg/mL) diluted in 450 μL of water. The obtained mixtures were incubated at room temperature for 5 min. The reactions were started by adding 200 μL of 5 mM of 3-(2-pyridyl)-5,6-bis (4-phenyl-sulfonic acid)-1,2,4-triazine (ferrozine). The mixtures were then strongly shaken and were left to stand at room temperature for 10 min. Similarly, the control tube was prepared, replacing the sample with distilled water. EDTA was used as the positive control.

The solutions absorbance was measured at 562 nm, and the inhibition percentage of ferrozine-Fe^2+^ complex formation was calculated as follows:

$$\mathrm{Metal}\;\mathrm{chelating}\;\mathrm{activity}\;\%\;\mathrm{Ab}=(\frac{AC+AB-AS}{\mathrm{AC}})\;\times \;100$$

where AC, AB and AS are the control absorbance, the blank, and the sample reaction tubes, respectively.

### 3.6. Antimicrobial Activities

#### 3.6.1. Microorganisms

The bacterial strains were divided into 5 Gram-positive (*S. epidermidis* CIP 106510, *E. feacalis* ATCC 29212, *M. luteus* NCIMB 8166, *B. cereus* ATCC 11778, *L. monocytogenes* ATCC19115) and 2 Gram-negative bacteria (*E. coli* ATCC 35218, *S. typhimurium* LT2 DT104). The fungal species belonged to 4 *Candida* strains (*C. albicans* ATCC 90028; *C. glabrata* ATCC 90030; *C. parapsilosis* ATCC 22019; *C. krusei* ATCC 6258). These strains were chosen for their ability to cause serious human infections.

#### 3.6.2. Disc-Diffusion Assay

The antimicrobial activity testing was done according to the protocol described by Ben Bnina et al. [55]. For the experiments, a small amount of the microorganism’s working stocks was added to a tube containing 9 mL of Mueller–Hinton broth (for bacteria) and Sabouraudchloramphenicol broth (for yeast strains). The mixture was then incubated at 37 °C for 18 to 24 h. The resulting overnight cultures were utilized to assess the antimicrobial activity of the extract in this study. The optical density was adjusted to 0.5 McFarland turbidity standards using a DENSIMAT (Biomérieux^®^, Marcy-l’Étoile, France). The respective bacteria and fungi were streaked onto MH or SB agar plates using a sterile swab.

Sterile filter discs with a diameter of 6 mm, made from Whatman paper No. 3, were soaked in an extract solution with a concentration of 150 mg/mL. These impregnated discs were then placed onto the appropriate agar media, which included the Sabouraud chloramphenicol broth (SB) and the Mueller–Hinton broth (MH). Gentamycin (10 μg/disc) and Amphotericin B (20 μg/disc) were used as positive reference standards. They were employed to gauge the susceptibility of a particular strain or isolate to each of the tested microbial species.

Following an incubation period at 37 °C lasting 18 to 24 h, the diameter of the inhibition zone around each disc was measured using a 1 mm flat rule. These measurements were interpreted according to the guidelines provided by the Committee of the French Society of the Antibiogram [56]. The dishes were kept in an incubator at 37 °C for 18 to 24 h for the microbial strains to develop. The extent of the inhibition zones surrounding each disc served as a measure of the antimicrobial activity. Every experiment was conducted three times (triplicate), and the average diameter of the inhibition zones was recorded.

#### 3.6.3. Micro-Well Determination of MIC, MBC and MFC

Minimal inhibition concentration (MIC), minimal bactericidal concentration (MBC), and minimal fungicidal concentration (MFC) values were determined, as described by Zanati et al. [57], for all bacterial and fungal strains used in this study. A 100 μL aliquot from stock solutions of the extract was added into the first wells. Then, 100 μL from the serial dilutions were transferred into eleven consecutive wells. The last well containing 195 μL of the nutrient broth without the extract and 5 μL of the inoculum on each strip was used as the negative control. The final volume in each well was 200 μL. The plates were incubated at 37 °C for 18–24 h. The extract tested in this study was screened two times against each organism. The MIC (Minimal Inhibition Concentration) value was defined as the lowest concentration of the compounds to inhibit the growth of the microorganisms. The MBC (Minimal Bactericidal Concentration) and MFC (Minimal Fungicidal Concentration) values were interpreted as the highest dilution (lowest concentration) of the sample, which showed clear fluid with no development of turbidity and without visible growth. Furthermore, we determined the MBC/MIC and MFC/MIC ratios to better understand the potential bacteriostatic or bactericidal effects of our extract. Each test was carried out on a single occasion. According to the categorization provided by Schaechter et al. [41] and Soro et al. [42], if the ratio surpasses 4, it signifies a bacteriostatic or fungistatic effect. Conversely, if the ratio is 4 or lower, it suggests a bactericidal or fungicidal effect.

### 3.7. Statistical Analysis

The experiments (antioxidant and antimicrobial activities) were conducted in triplicates, and the average values were calculated using the SPSS 25.0 statistical package for Windows. Differences in means were analyzed using Duncan’s multiple-range tests with a 95% confidence interval (*p* ≤ 0.05).

## 4. Conclusions

Overall, the Tunisian *C. annuum* seed cultivar was proven to be abundant in various secondary metabolites endowed with considerable antioxidant potential as well as an important antimicrobial activity against seven bacterial and four fungal strains. The identified compounds in the hydro-methanolic seed extract of *C. annuum* belonged to various phytochemical classes, such as organic acids, phenolic compounds, capsaicinoids, capsianosides, fatty acids, amino acids, sphingolipids, and steroids. The combined presence of these bioactive compounds in this extract likely contributes to its broad-spectrum antimicrobial efficacy against both bacterial and fungal strains. Further research and studies may be needed to determine the exact mechanisms of action and potential applications of this extract as a natural antimicrobial agent.

## Figures and Tables

**Table 1:** RP-HPLC-DAD-QTOF-MS/MS analysis of the Tunisian *C. annuum* hydro-methanolic seed extract.

No	Proposed Identity	Class	T_R_ (min)	Ion Type	HRMS (*m*/*z*)	MF	HRMS/MS (*m*/*z*)
1	Galactonic/gluconic acid	Organic acid	1.2	[M−H]^−^	195.0563	C_6_H_12_O_7_	177.0444, 159.0358, 129.0233
2	*N*-Fructosyl(iso)leucine	Amino acid	1.8	[M+H]^+^	294.1539	C_12_H_23_NO_7_	258.1321, 230.1388, 211.0604, 144.0990, 114.0984
3	Citric acid	Organic acid	2.0	[M−H]^−^	191.0241	C_6_H_8_O_7_	173.0106, 154.9924, 129.0135, 111.089
4	Hydroxybenzoic acid-*O*-hexoside	Phenolic acid	10.2	[M−H]^−^	299.0845	C_13_H_16_O_8_	239.0589, 179.0409, 137.0301
5	Vanillic acid-*O*-hexoside	Phenolic acid	11.6	[M−H]^−^	329.0943	C_14_H_18_O_9_	209.0571, 167.0382, 125.0279
6	Sinapic acid-*O*-hexoside	Phenolic acid	15.8	[M−H]^−^	385.1938	C_17_H_22_O_10_	223.1360, 168.0088, 153.0899
7	Ferulic acid-*O*-hexoside	Phenolic acid	16.5	[M−H]^−^	355.1106	C_16_H_20_O_9_	235.0688, 217.0575, 193.0542, 175.0440
8	2-Aminododecane-1,3-diol	Sphingolipid	17.7	[M+H]^+^	218.2116	C_12_H_27_NO_2_	200.2046, 156.1870
9	Tetradecaphytosphingosine	Sphingolipid	19.3	[M+H]^+^	262.2377	C_14_H_31_NO_3_	200.1996, 109.0575
10	Luteolin-*O*-pentoside-C-hexoside	Flavonoid	23.2	[M−H]^−^	579.1437	C_26_H_28_O_16_	447.0987, 327.0554, 297.0437, 285.0445, 151.0065
11	Quercetin-*O*-deoxyhexoside	Flavonoid	25.4	[M−H]^−^	447.1011	C_21_H_20_O_11_	300.0480, 271.0443, 255.0404, 179.0086, 163.0230, 151.0156
12	Capsicoside A	Saponin	26.2	[M−H]^−^	1421.6593	C_63_H_106_O_35_	1259.5962, 1097.4411, 935.4233, 773.4179, 663.2902
13	Tetradecasphinganine	Sphingolipid	26.5	[M+H]^+^	246.2417	C_14_H_31_NO_2_	228.2353, 163.0689, 106.0814
14	(Epi)catechin	Flavonoid	27.0	[M−H]^−^	289.1038	C_15_H_14_O_6_	245.0865, 205.0511, 179.0332
15	Hexadecaphytosphingosine I	Sphingolipid	27.4	[M+H]^+^	290.2678	C_16_H_35_NO_3_	228.2282, 102.0922
16	Capsianoside III	Diterpene	28.1	[M−H]^−^	1099.5317	C_50_H_84_O_26_	937.4708, 793.4166, 775.4363, 629.3666, 479.3652
17	Protodegalactotigonin	Saponin	29.9	[M−H]^−^	1213.6011	C_56_H_94_O_28_	1081.5401, 919.5061, 757.4474
18	Capsianoside IX	Capsianoside	30.1	[M−H]^−^	937.4775	C_44_H_74_O_21_	791.4181, 629.3639, 483.2961, 467.2926
19	Capsianoside XV	Capsianoside	31.5	[M−H]^−^	1099.5310	C_50_H_84_O_26_	937.4766, 775.4302, 629.3895, 467.2933
20	Quercetin	Flavonoid	31.9	[M−H]^−^	301.0388	C_15_H_10_O_7_	273.0376, 178.9983, 107.0112
21	Hexadecasphinganine	Sphingolipid	32.8	[M+H]^+^	274.2736	C_16_H_35_NO_2_	212.2237, 106.0752, 102.0930
22	Capsianoside II	Capsianoside	33.1	[M−H]^−^	1083.5379	C_50_H_84_O_25_	937.4759, 921.4850, 775.4192, 757.4132, 611.3466
23	Phytosphingosine I	Sphingolipid	33.3	[M+H]^+^	318.3009	C_18_H_39_NO_3_	256.2627, 102.0818
24	Hexadecaphytosphingosine II	Sphingolipid	34.0	[M+H]^+^	290.2701	C_16_H_35_NO_3_	242.2430, 171.0999, 122.0756
25	Capsianoside VIII	Capsianoside	34.5	[M−H]^−^	1083.5348	C_50_H_84_O_25_	937.4877, 921.4761, 775.4152, 757.4161, 629.3696, 467.3276
26	Luteolin	Flavonoid	35.8	[M−H]^−^	285.0407	C_15_H_14_O_6_	267.0298, 258.0453, 151.0030
27	Trihydroxyoctadecenoic acid I	Fatty acid	36.8	[M−H]^−^	329.2410	C_18_H_34_O_5_	312.2237, 293.2175, 201.1169, 171.1047
28	Sphinganine	Sphingolipid	37.9	[M+H]^+^	302.3064	C_18_H_39_NO_2_	106.0868
29	Trihydroxyoctadecenoic acid II	Fatty acid	38.7	[M−H]^−^	329.2411	C_18_H_34_O_5_	227.1443, 211.15406, 171.1143
30	Phytosphingosine II	Sphingolipid	39.0	[M+H]^+^	318.3018	C_18_H_39_NO_3_	300.2913, 122.0825
31	Hydroxyoctadecatrienoic acid I	Fatty acid	40.3	[M−H]^−^	293.1838	C_18_H_30_O_3_	236.1074, 221.1527
32	Nordihydrocapsaicin	Capsaicinoid	42.4	[M+H]^+^	294.2072	C_17_H_27_NO_3_	170.1505, 137.0589, 123.1110
33	Hydroperoxyoctadecadienoic acid I	Fatty acid	42.4	[M−H]^−^	311.2306	C_18_H_32_O_4_	293.1986, 275.2096, 256.1854, 223.1707, 207.1317
34	Capsaicin	Capsaicinoid	43.2	[M+H]^+^	306.2069	C_18_H_27_NO_3_	182.1524, 170.1515, 153.1255, 137.0579
35	Dihydroxyoctadecenoic acid I	Fatty acid	45.1	[M−H]^−^	313.2456	C_18_H_34_O_4_	250.5000, 183.1416, 129.0978
36	Dihydroxyoctadecenoic acid II	Fatty acid	45.9	[M−H]^−^	313.2466	C_18_H_34_O_4_	297.2344, 278.2035, 241.1174, 201.1164
37	Dihydrocapsaicin	Capsaicinoid	46.5	[M+H]^+^	308.2229	C_18_H_29_NO_3_	184.1668, 137.0587, 122.0350
38	*N*-Hydroxy arachidonoyl amine	Sphingolipid	48.3	[M+H]^+^	320.2581	C_20_H_33_NO_2_	262.1713, 123.0397
39	Hydroxyoctadecadienoic acid I	Fatty acid	52.6	[M−H]^−^	295.2360	C_18_H_32_O_3_	277.2189, 195.1437
40	Hydroxyoctadecatrienoic acid I	Fatty acid	53.4	[M−H]^−^	293.2200	C_18_H_30_O_3_	275.1981, 235.1642
41	Hydroxyoctadecatrienoic acid II	Fatty acid	54.2	[M−H]^−^	293.2192	C_18_H_30_O_3_	195.1651, 171.1170
42	Hydroxyoctadecatrienoic acid III	Fatty acid	54.9	[M−H]^−^	293.2203	C_18_H_30_O_3_	236.1141, 185.1146
43	Trihydroxyoctadecanoic acid	Fatty acid	56.1	[M−H]^−^	331.2124	C_18_H_36_O_5_	295.2367, 226.5369
44	Hydroxyoctadecadienoic acid II	Fatty acid	57.5	[M−H]^−^	295.2357	C_18_H_32_O_3_	277.2233, 195.1459, 171.1072, 123.1208
45	Hydroxyoctadecadienoic acid III	Fatty acid	59.3	[M−H]^−^	295.2354	C_18_H_32_O_3_	249.2159, 141.1321

**Table 2:** Polyphenol content (mg GAE/g DW), flavonoid content (mg QE/g DW) and antioxidant activities (DPPH test, IC_50_), reducing power (FRAP, EC_50_), chelating power (CP, IC_50_) of the Tunisian *C. annuum* hydro-methanolic seed extract.

	DPPH	FRAP	CP	Polyphenol Content	Flavonoid Content
Extract	45.0 ± 2.0 ^a^	61.3±0.6 ^a^	79.0 ± 1.0 ^a^	193.7 ± 3.1	25.1 ± 1.1
BHT	11.5 ± 0.6 ^b^	23.0 ±1.0 ^c^	-	-	-
Vitamin C	-	37.0 ±2.0 ^b^	-	-	-
EDTA	-	-	32.5 ± 1.3 ^b^	-	-

Means (three replicates) followed by at least one same letter are not significantly different at *p* < 0.05.

**Table 3:** Zones of growth inhibition (IZ mm±SD), minimal inhibition concentration (MIC mg/mL), minimal bactericidal concentration (MBC mg/mL) and ratios (MBC/MIC and MFC/MIC) showing quantitative antimicrobial activity for the Tunisian *C. annuum* hydro-methanolic seed extract against human pathogenic bacteria and candida compared to that of the positive standard antibiotic/antifungal (gentamycin/amphotericin B).

Microorganisms	Extract				Antibiotic/Antifungal			
					Gentamycin			
Bacteria Strains	IZ ^a^	MIC	MBC	MBC/MIC	IZ ^b^	MIC	MBC	MBC/MIC
*S. epidermidis* CIP 106510	10.83 ± 0.76 ^b^	1.875	3.750	2 (Bactericidal)	21.33 ± 0.58 ^efg^	0.009	0.039	4 (Bactericidal)
*M. luteus* NCIMB 8166	10.33 ± 0.57 ^bc^	0.938	1.875	2 (Bactericidal)	27.67 ± 1.53 ^a^	0.004	0.019	4 (Bactericidal)
*E. feacalis* ATCC 29212	9.33 ± 0.57 ^c^	0.938	3.750	4 (Bactericidal)	26.00 ± 1.00 ^b^	0.004	0.019	4 (Bactericidal)
*B. cereus* ATCC 11778	9.00 ± 1.00 ^c^	1.875	3.750	2 (Bactericidal)	26.00 ± 1.00 ^b^	0.004	0.039	8 (Bacteriostatic)
*E.coli* ATCC 35218	11.66 ± 0.57 ^a^	1.875	7.500	2 (Bactericidal)	22.00 ±1.00 ^def^	0.009	0.039	4 (Bactericidal)
*L. monocytogenes* ATCC19115	11.00 ± 0.0 ^b^	1.875	3.750	4 (Bactericidal)	23.00 ± 0.0 ^cd^	0.019	0.078	4 (Bactericidal)
*S. typhimurium* LT2 DT104	12.00 ± 0.0 ^a^	1.875	3.750	2 (Bactericidal)	20.33 ± 0.57 ^g^	0.019	0.039	2 (Bactericidal)
Yeast strains					**Amphotericin B**			
*C. albicans* ATCC 90028	13.66 ± 0.57 ^a^	0.234	0.938	4 (Fungicidal)	18 ± 0.0 ^a^	0.078	0.310	4 (Fungicidal)
*C. glabrata* ATCC 90030	13.00 ± 1.00 ^a^	0.234	0.938	4 (Fungicidal)	16.33 ± 0.57 ^b^	0.009	0.078	8 (Fungistatic)
*C. parapsilosis* ATCC 22019	13.00 ± 0.0 ^a^	0.938	1.875	2 (Fungicidal)	17.33 ± 0.57 ^a^	0.039	0.078	2 (Fungicidal)
*C. krusei* ATCC 6258	12.66 ± 0.57 ^ab^	0.234	0.938	4 (Fungicidal)	16 ± 0.0 ^b^	0.009	0.019	4 (Fungicidal)

SD: Standard deviation; IZ ^a^: Inhibition zone in diameter (mm) around the discs (6 mm) impregnated with 150 mg/mL of hydro-methanolicextract; IZ ^b^: Inhibition zone in diameter (mm) of gentamycin (20 μg/disc) and amphotericin B (20 μg/disc) were used as positive reference standards antibiotic discs. Means (three replicates) followed by at least one same letter are not significantly different at *p* < 0.05.

## Data Availability

The data presented in this study are available on request from thecorresponding author.

## Sample Availability

Samples of the compounds are available from the authors.

## References

[1] Bosland P.W., Votava E.J. (2000). Peppers: Vegetable and Spice Capsicums.

[2] da Veiga V.F., Wiedemann L.S.M., de Araujo C.P., da Silva Antonio A. (2022). Chapter 1: Origin and Evolution of *Capsicum*. Chemistry and Nutritional Effects of Capsicum.

[3] Dimitrios B. (2006). Sources of natural phenolic antioxidants. Trends Food Sci..

[4] Olatunji T.L., Afolayan A.J. (2019). Comparative quantitative study on phytochemicalcontents and antioxidant activities of *Capsicum annuum* L. and *Capsicum frutescens* L.. Sci. World J..

[5] Lahbib K., Bnejdi F., ElGazzah M. (2013). Selection of pepper parent from a collection of *Capsicum annuum* landraces on genetic diversity. J. Plant Breed. Crop Sci..

[6] Zhani K., Hamdi W., Sedraoui S., Fendri R., Lajim O., Hannachi C. (2015). Agronomic evaluation of Tunisian accessions of chili pepper (*Capsicum frutescens* L.). Int. Res. J. Eng. Technol..

[7] Zhani K., Hamdi W., Sedraoui S., Fendri R., Lajim O., Hannachi C. (2015). A comparative study of morphological characterization of Tunisian accessions of Chili pepper (*Capsicum frutescens* L.). Int. Res. J. Eng. Technol..

[8] Loizzo M.R., Pugliese A., Bones M., Menichini F., Tundis R. (2015). Evaluation of chemical profile and antioxidant activity of twenty cultivars from *Capsicum annuum*, *Capsicum baccaum*, *Capsicum chacoense* and *Capsicum chinense*: A comparison between fresh and processed peppers. Food Sci. Technol..

[9] Materska M. (2014). Bioactive phenolics of fresh and freeze-dried sweet and semi-spicy pepper fruits (*Capsicum annuum* L.). J. Funct. Foods.

[10] Pugliese A., Loizzo M.R., Tundis R., O’Callaghan Y., Menichini F., O’Brie N., Galvin K. (2013). The effect of domestic processing on the content and bioaccessibility of carotenoids from chili peppers (*Capsicum* species). Food Chem..

[11] Halikowski Smith S. (2015). In the shadow of a pepper-centric historiography: Understanding the global diffusion of capsicums in the sixteenth and seventeenth centuries. J. Ethnopharmacol..

[12] Materska M., Konopacka M., Rogolinsk J., Slosarek K. (2015). Antioxidant activity and protective effects against oxidative damage of human cells induced by X-radiation of phenolic glycosides isolated from pepper fruits *Capsicum annuum* L.. Food Chem..

[13] Howard L.R., Wildman R.E.C., Wildman R.E.C. (2007). Antioxidant vitamin and phytochemical content of fresh and processed pepper fruit (*Capsicum annuum*). Handbook of Nutraceuticals and Functional Foods.

[14] Naczk M., Shahidi F. (2004). Extraction and Analysis of Phenolics in Food. J. Chromatogr..

[15] Crozier A., Jaganath I.B., Clifford M.N., Crozier A., Clifford M.N., Ashihara H. (2006). Plant Secondary Metabolites: Occurrence, Structure and Role in the Human Diet.

[16] Pietta P., Minoggio M., Bramati L., Rahman A. (2003). Studies in Natural Products Chemistry.

[17] Tundis R., Loizzo M.R., Menichini F., Bonesi M., Conforti F., Statti G., De Luca D., de Cindio B., Menichini F. (2011). Comparative study on the chemical composition, antioxidant properties and hypoglycaemic activities of two *Capsicum annuum* L. cultivars (*Acuminatum* small and *Cerasiferum*). Plant Foods Hum. Nutr..

[18] Park J.-H., Jeon G.-I., Kim J.-M., Park E. (2012). Antioxidant activity and antiproliferative action of methanol extracts of 4 different colored bell peppers (*Capsicum annuum* L.). Food Sci.Biotechnol..

[19] Koffi-Nevry R., Kouassi K., Nanga Z., Koussémon M., Loukou G. (2012). Antibacterial activity of two bell pepper extracts: *Capsicum annuum* L. and *Capsicum frutescens*. Int. J. Food Prop..

[20] Sree Sandhya M.V., Vijayakumar N. (2016). Comparative Study on Antimicrobial Activity of Eight Capsicum Species—A novel Therapeutic compound. Indian J. Res..

[21] Koffi A.C., Koffi A.R., Kossonou Y.K., et Koffi-Nevry R. (2021). Activitéantimicrobienne et composition phytochimiqued’extraits de piment “*Capsicum* sp.”. ” Pharm. Méd. Tradit. Afr..

[22] Leng Z., Zhong B., Wu H., Liu Z., Rauf A., Bawazeer S., Suleria H.A.R. (2022). Identification of Phenolic Compounds in Australian-Grown Bell Peppers by Liquid Chromatography Coupled with Electrospray Ionization-Quadrupole-Time-of-Flight-Mass Spectrometry and Estimation of Their Antioxidant Potential. ACS Omega.

[23] Pereira C., Barros L., Carvalho A.M., Ferreira I.C.F.R. (2013). Use of UFLC-PDA for the analysis of organic acids in thirty-five species of food and medicinal plants. Food Anal. Methods.

[24] Abidi J., Ammar S., Ben Brahim S., Skalicka-Woźniak K., GhrabiGammar Z., Bouaziz M. (2019). Use of ultra-high-performance liquid chromatography coupled with quadrupole-time-of-flight mass spectrometry system as valuable tool for an untargeted metabolomic profiling of *Rumextunetanus* flowers and stems and contribution to the antioxidant activity. J. Pharm Biomed. Anal..

[25] Denev P., Todorova V., Ognyanov M., Georgiev Y., Yanakieva I., Tringovska I., Grozeva S., Kostova D. (2019). Phytochemical composition and antioxidant activity of 63 Balkan pepper (*Capsicum annuum* L.) accessions. J. Food Meas. Charact..

[26] Moreno-Ramírez Y.R., Martínez-Ávila G.C.G., González-Hernández V.A., Castro-López C., Torres-Castillo J.A. (2018). Free Radical-Scavenging Capacities, Phenolics and Capsaicinoids in Wild Piquin Chili (*Capsicum annuum* var. *Glabriusculum*). Molecules.

[27] del Aguiar A.C., da Fonseca Machado A.P., FigueiredoAngolini C., de Morais D.R., Baseggio A.M., NogueiraEberlin M., Maróstica Junior M.R., Julian M. (2019). Sequential high-pressure extraction to obtain capsinoids and phenolic compounds from biquinho pepper (*Capsicum chinense*). J. Supercrit. Fluids.

[28] Morales-Soto A., Gómez-Caravaca A.M., García-Salas P., Segura-Carretero A., Fernández-Gutiérrez A. (2013). High-performance liquid chromatography coupled to diode array and electrospray time-of-flight mass spectrometry detectors for a comprehensive characterization of phenolic and other polar compounds in three pepper (*Capsicum annuum* L.) samples. Food Res. Int..

[29] Kelebek H., Sevindik O., Uzlasir T., Selli S. (2020). LC-DAD/ESI MS/MS characterization of fresh and cooked Capia and Aleppo red peppers (*Capsicum annuum* L.) phenolic profiles. Eur. Food Res. Technol..

[30] Santos L.S., Fernandes C.C., Santos L.S., de Deus I.P.B., de Sousa T.L., Miranda M.L.D. (2021). Ethanolic extract from *Capsicum chinense* Jacq. ripe fruits: Phenolic compounds, antioxidant activity and development of biodegradable films. Food Sci. Technol.Camp..

[31] Stöggl W.M., Huck C.W., Bonn G.K. (2004). Structural elucidation of catechin and epicatechin in sorrel leaf extracts using liquid-chromatography coupled to diode array-, fluorescence-, and mass spectrometric detection. J. Sep. Sci..

[32] Chang C., Wu R. (2011). Quantification of (+)-catechin and (−)-epicatechin in coconut water by LC–MS. Food Chem..

[33] Jeong W.Y., Jin J.S., Cho Y.A., Lee J.H., Park S., Jeong S.W., Kim Y.H., Lim C.S., Abd El-Aty A.M., Kim G.S. (2011). Determination of polyphenols in three *Capsicum annuum* L. (bell pepper) varieties using high-performance liquid chromatographytandem mass spectrometry: Their contribution to overall antioxidant and anticancer activity. J. Sep. Sci..

[34] Schelz Z., Molnár J., Fogliano V., Ferracane R., Pernice R., Shirataki Y., Motohashi N. (2006). Qualitative Analysis of MDR-reversing Anastasia Black (Russian Black Sweet Pepper, *Capsicum annuum*, Solanaceae) Extracts and Fractions by HPLC and LC-MS-MS Methods. In Vivo.

[35] Chilczuk B., Marciniak B., Stochmal A., Pecio Ł., Kontek R., Jackowska I., Materska M. (2020). Anticancer Potential and Capsianosides identification in Lipophilic Fraction of Sweet Pepper (*Capsicum annuum* L.). Molecules.

[36] Menezes R.d.P., Bessa M.A.d.S., Siqueira C.d.P., Teixeira S.C., Ferro E.A.V., Martins M.M., Cunha L.C.S., Martins C.H.G. (2022). Antimicrobial, Antivirulence, and Antiparasitic Potential of *Capsicum chinense* Jacq. Extracts and Their Isolated Compound Capsaicin. Antibiotics.

[37] Guevara L., Domínguez-Anaya M.Á., Ortigosa A., González-Gordo S., Díaz C., Vicente F., Corpas F.J., Pérez del Palacio J., Palma J.M. (2021). Identification of Compounds with Potential Therapeutic Uses from Sweet Pepper (*Capsicum annuum* L.) Fruits and Their Modulation by Nitric Oxide (NO). Int. J. Mol. Sci..

[38] Cervantes-Hernández F., Ochoa-Alejo N., Martínez O., Ordaz-Ortiz J.J. (2022). Metabolomic Analysis Identifies Differences between Wild and Domesticated Chili Pepper Fruits During Development (*Capsicum annuum* L.). Front. Plant Sci..

[39] Yahara S., Ura T., Sakamoto C., Nohara T. (1994). Steroidal glycosides from *Capsicum annuum*. Phytochemistry.

[40] Chouaib H., Ayadi I., Zouari S., Fakhfakh N., Zaidi S., Zouari N. (2012). Effect of phenological stage and geographical location on antioxydant activities of Tunisian horehound: *Marrubiumvulgare* L. (Lamiaceae). J. Biol. Act. Prod. Nat..

[41] Schaechter M., Medoff G., Barry I., Eisenstein B.I. (1999). Microbiologie et PathologieInfectieuse.

[42] Soro D., Kone M.W., Kamanz I.K. (2010). Evaluation des activitésantimicrobiennes et anti-radicauxlibres de quelquestaxonsbioactifs de Cote d’Ivoire. Eur. J. Sci. Res..

[43] Elif A., Erdoğan E. (2020). Antimicrobial activity of citric acid. Eur. J. For. Sci..

[44] Munirah F.A. (2023). The synergistic effect of capsicum aqueous extract (*Capsicum annuum*) and chitosan against multidrug-resistant bacteria. J. King Saud Univ. Sci..

[45] Sidra K., Zaheer H.S., Saira R., Naveed A., Shumaila Islam M., Akram R., Shahzad N. (2020). Antimicrobial activity of citric acid functionalized iron oxide nanoparticles–Superparamagnetic effect. Ceram Int..

[46] Pascal N.M., Serena M., Julienne N., Davide T., Emilio S. (2019). Phenolic compounds profile of water and ethanol extracts of *Euphorbia hirta* L.leaves showing antioxidant and antifungal properties. S. Afr. J. Bot..

[47] Egle V., Ilona J., Michail S., Ernesta A., Paulius M., Andrius S., Naglis M. (2020). Advances and Prospects of Phenolic Acids Production, Biorefinery and Analysis. Biomolecules.

[48] Marini E., Magi G., Mingoia M., Pugnaloni A., Facinelli B. (2015). Antimicrobial and Anti-Virulence Activity of Capsaicin against Erythromycin-Resistant, Cell-Invasive Group a Streptococci. Front. Microbiol..

[49] Ammar S., Abidi J., Vlad Luca S., Boumendjel M., Skalicka-Woźniak K., Bouaziz M. (2020). Untargeted metabolite profiling and phytochemical analysis based on RP-HPLC-DAD-QTOF-MS and MS/MS for discovering new bioactive compounds in *Rumexalgeriensis* flowers and stems. Phytochem. Anal..

[50] Gargouri B., Ammar S., Zribi A., Mansour A.B., Bouaziz M. (2013). Effect of growing region on quality characteristics and phenolic compounds of Chemlali extra-virgin olive oils. Acta Physiol. Plant..

[51] Mouhamadi N., Meraghni M., Necib A., Jelaiel L., El Arbi M., Bouaziz M. (2023). Comparative Study on Chemical Composition of Green and Black Table Olives Brines of the Endemic “Sigoise” Cultivar: Recovery of high—Added Values Compounds. Chem. Biodivers..

[52] Bouaziz M., Jemai H., Khabou W., Sayadi S. (2010). Oil content, phenolic profiling and antioxidant potential of Tunisian olive drupes. J. Sci. Food Agric..

[53] Yildirim A., Mavi A., Oktay M., Kara A.A., Algur Ö.F., Bilaloglu V. (2000). Comparison of antioxidant and antimicrobial activities of tilia (*Tiliaargentea* Desf Ex DC), sage (*Salvia triloba* L.) and black tea (*Camellia sinensis*) extracts. J. Agric. Food Chem..

[54] Dhouibi I., Flamini G., Bouaziz M. (2023). Comparative Study on the Essential Oils Extracted from Tunisian Rosemary and Myrtle: Chemical Profiles, Quality, and Antimicrobial Activities. ACS Omega.

[55] Ben Bnina E., Hajlaoui H., Chaieb I., Daami-Remadi M., Ben Said M., Ben Jannet H. (2019). Chemical composition, antimicrobial and insecticidal activities of the tunisian *Citrus aurantium* essential oils. Czech J. Food Sci..

[56] Cavallo J.D., Chardon H., Chidiac C., Choutet P., Courvalin P., Dabernat H., Drugeon H., Dubreuil L., Goldstein F., Jarlier V. (2006). Comité de l’antibiogramme de la sociétéFrançaise de Microbiologie. Communiqué.

[57] Znatia M., Jabrane A., Hajlaoui H., Harzallah-Skhiri F., Bouajila J., Casanova J., Ben Jannet H. (2012). Chemical Composition and in vitro Evaluation of Antimicrobial and Anti-acetylcholinesterase Properties of the Flower Oil of *Ferula lutea*. Nat. Prod. Commun..

